# Hidden Behind Ascites: An Atypical Pediatric Presentation of T‐Cell Acute Lymphoblastic Leukemia—A Case Report

**DOI:** 10.1002/ccr3.72858

**Published:** 2026-06-02

**Authors:** Kainat Shaikh, Zahra Anas, Samia Rajput, Alizeh Zaib, Ayesha Saleem, Md Ariful Haque

**Affiliations:** ^1^ Department of Pediatrics Dr Ruth KM Pfau Civil Hospital Karachi Pakistan; ^2^ Department of Pediatrics DOW University of Health Sciences Karachi Pakistan; ^3^ Department of Medicine Dr Ruth KM Pfau Civil Hospital Karachi Pakistan; ^4^ Department of Medicine DOW University of Health Sciences Karachi Pakistan; ^5^ Department of Internal Medicine Liaquat College of Medicine and Dentistry Karachi Pakistan; ^6^ Voice of Doctors Research School Dhaka Bangladesh; ^7^ Department of Public Health Atish Dipankar University of Science and Technology Dhaka Bangladesh

**Keywords:** acute lymphoblastic leukemia, ascites, pediatric, T‐cell

## Abstract

This case describes a child with T‐cell acute lymphoblastic leukemia (T‐ALL) presenting atypically with ascites and bilateral pedal edema, initially suggesting autoimmune hepatitis. The diagnosis was established only after flow cytometry of ascitic fluid. It underscores that pediatric malignancies may present atypically and highlights the importance of a broad, flexible diagnostic approach to avoid delays in life‐saving treatment.

## 1 Introduction

Acute Lymphoblastic Leukemia (ALL) is the most prevalent childhood malignancy. Approximately 25% of pediatric neoplasia diagnoses are related to ALL. Acute Lymphoblastic leukemia can be classified according to the lymphoblast cells, such as B‐cell and T‐cell ALL. T‐cell ALL (T‐ALL) is characterized by the proliferation of uncontrolled immature T‐lymphocytes. Although 85% of ALL cases arise from the B‐cell lineage, T‐ALL has unusual morphology and presentation. Additionally, it presents in 60% of children and adolescents as a mediastinal mass; 50% with only a high white blood cell count; and 10% with Central Nervous system involvement. Involvement of extramedullary organs, such as the liver and spleen, can occur. The involvement of multiple organs in this tumor can mislead different diagnoses and atypical clinical presentations [1, 2, 3, 4, 5].

The association of autoimmune disease such as SLE, Rheumatoid Arthritis, autoimmune hepatitis is linked to Leukemia significantly after a recent systematic review study conducted by Ventela et al. This emphasizes the shared etiology between these two entirely different disease processes and their correlation [6].

This case report details the course of a pediatric patient diagnosed with T‐cell Acute Lymphoblastic Leukemia (T‐ALL) with an unusual initial presentation. It includes the diagnostic challenges encountered and the importance of advanced imaging modalities.

## 2 Case History/Examination

A 12‐year‐old girl presented with a 2‐month history of persistent and progressive abdominal swelling with fever for 2 days at a tertiary care hospital. Her parents recalled that the fever was high‐grade, documented up to 102°F, not associated with rigors or chills, and partially responsive to antipyretics. Fever was not associated with seizures, rashes, vomiting, or weight loss. For 2 months, her abdomen began to enlarge, which progressed to facial and bilateral pedal swelling that slowly progressed to her shins. A few weeks before admission, her periorbital puffiness worsened, and she became breathless with even minimal exertion, which led her parents to seek urgent medical care. There was no history of joint pain, jaundice, gastrointestinal bleeding, hematuria, frothy urine, or cough. She had no significant history of previous hospitalizations, was up to date with immunizations, and lived in a crowded household with a history of treated pulmonary tuberculosis.

On examination, she had general pallor and fatigue with a sound mental status and was oriented to time, place, and person. She was febrile at a temperature of 38.5°C, with a pulse of 139 beats per minute indicating tachycardia, and an increased respiratory rate of 30 breaths per minute. Abdominal examination revealed marked distension and increased abdominal girth measuring 70 cm, with distended veins visible over the skin of the abdomen and an everted umbilicus. Hepatosplenomegaly was represented by liver extending 5 cm below the right costal margin with a span of 20 cm, and the splenomegaly by palpable spleen 13 cm below the left costal margin. The margins and consistency of the hepatosplenomegaly were firm and smooth. Ascites were detected, confirmed by fluid thrill and shifting dullness. She had generalized edema, which was most pronounced in the lower limbs, and a solitary 1 × 1 cm anterior cervical lymph node was palpable. Cardiovascular examination revealed a hyperdynamic apex beat; however, heart sounds were normal, and no murmurs were detected. Respiratory findings included increased work of breathing with equal and bilateral air entry. Neurological examination was normal, and her Glasgow Coma Scale score was 15/15.

## 3 Differential Diagnosis, Investigation and Treatment

During admission, initial blood investigations revealed anemia with hemoglobin at 9.2 g/dL, MCV of 89 fL, a total leukocyte count of 5.7 × 10^9^/L with 79% lymphocytes and 13% neutrophils, and platelets at 155 × 10^9^ cells/L. Her ESR was markedly elevated at 62 mm/h, and her serum albumin level was reduced to 2.8 g/dL. Liver Function Tests showed an elevation in alkaline phosphatase level to 282 U/L, although alanine aminotransferase was only 8 U/L. Ferritin was markedly raised at 785 ng/mL, and a corrected retic count of 2.8%. A peripheral smear showed anisopoikilocytosis with teardrop and elliptical cells, as well as low platelet count, raising suspicion for a marrow infiltrative process (Table 1).

**TABLE 1:** Various lab parameters on admission.

Hematology	Patient's labs	Normal range	Units
Hb	9.2	11–17.3	g/dL
MCV	89.7	90.4–128	fL
MCH	32.1	26–41.1	pg
MCHC	28.7	25.8–33.6	g/dL
HCT	35.4	35.4–56.5	%
TLC	5.7	3.1–21.6	×10E9/L
Lymphocytes	79	15–75	%
Neutrophils	13	15–78	%
Platelets	155	152–472	×10E9/L
Rectic count	(Cor. 2.68)		
G6PD	Not deficient		
UCS	No growth		
Blood Culture	No growth		
LDH	184		
Ferritin	785		
Ascitic Fluid analysis	Appearance: slightly turbid, Color: yellow, protein: 28, Albumin: 1.4, amylase 1, RBC: 2000, WBC: 733, Polymorphs: 733, lymphocytes: 98, Gram stain‐ve.		
Peripheral film	Anisocytosis, poikilocytosis, polychromasia, elliptical cells, tear drop cells, platelet low on film.		

Component		Normal range	Units
Basic metabolic profile			
BUN	2	21–40	mg/dL
Cr	0.4	0.61–1.24	mg/dL
Sodium	138	135–145	mEq/L
Potassium	3.9	3.5–5.5	mEq/L
Chloride	118	95–108	mEq/L
Calcium	8.2	7.5–10.0	mg/dL
Phosphate	2.9		mg/dL
Liver function tests			
ALT	8	130–831	U/L
ALP	282	130–83	U/L
Albumin	2.8	3.88–5.82	g/dl
Total Bilirubin	1.2	0.1–1.2	mg/dL
Direct Bilirubin	0.4	< 0.6	mg/dL
Indirect Bilirubin	1.6		mg/dL

Because of the positive ascites finding, an ascitic tap was performed. Ascitic fluid analysis demonstrated a yellow, slightly turbid fluid with a protein concentration of 28 g/dL, albumin of 1.4 g/dL, and a serum–ascites albumin gradient of 1.4, consistent with portal hypertension etiology. The fluid contained 733 white blood cells/μL, predominantly polymorphs and 98/μL lymphocytes and 2000**/**μL RBCs, but cultures and Gram staining were negative. Autoimmune screening revealed a strongly positive antinuclear antibody (ANA) at 1:640 and elevated IgG levels at 22.53 g/L, with negative anti‐smooth muscle, anti‐mitochondrial, and anti‐liver kidney microsomal antibodies, as shown in Table 2. The renal function was normal, as were the uric acid, ceruloplasmin, and lactate dehydrogenase levels. Screening for tuberculosis via the Mantoux test and gastric aspirate was negative.

**TABLE 2:** Autoantibody profile showing ANA positivity with elevated serum IgG, while other antibodies were negative.

Test	Result	Normal/positive range	Units
ANA	Positive 1:640	Normal: < 1:80 (negative) Positive: ≥ 1:80	Titer
ASMA	Negative	Normal: Negative Positive: ≥ 1:40	Titer
AMA	Negative	Normal: Negative Positive: ≥ 1:40	Titer
LKM	Negative	Normal: Negative Positive: ≥ 1:40	Titer
GPCS	Negative	Normal: Negative Positive: ≥ 1:40	Titer
Serum IgG levels	22.53	Normal: 7–16 g/L Elevated: > 16 g/L	g/L

**FIGURE 1:**
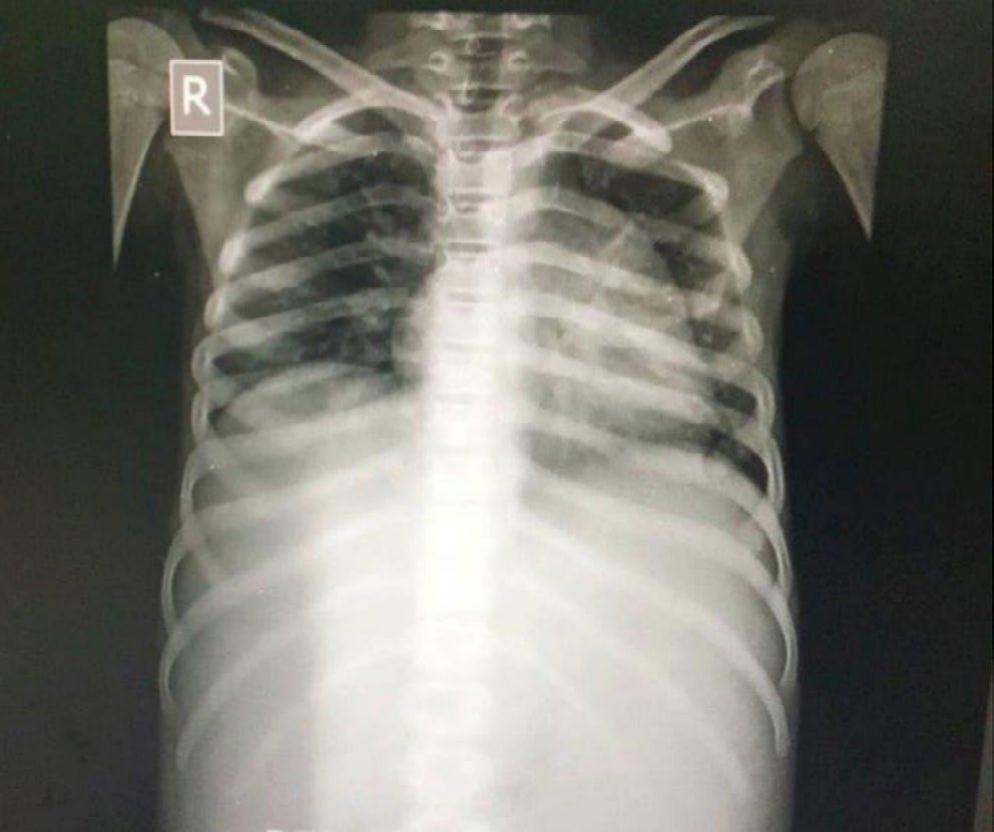
Chest X‐ray demonstrating pleural effusion with blunting of the costophrenic angle.

Radiological imaging, such as X‐ray, showed bilateral pleural effusions (Figure 1). Abdominal ultrasound confirmed massive hepatosplenomegaly, mild ascites, altered liver echotexture, a 19 cm, portal vein 0.9 cm, thick‐walled gallbladder surrounded by pericholecystic fluid, 14 cm enlarged spleen, and mild abdominal and pelvic ascites. CT imaging revealed para‐aortic lymphadenopathy, splenic vein attenuation with collaterals, and bilateral pleural effusions. Doppler studies ruled out portal vein thrombosis and Budd–Chiari syndrome (BCS). Echocardiography revealed mild pericardial effusion with a preserved ejection fraction (57%). The significant laboratory and imaging findings are summarized in Table 1.

Given the strongly positive ANA and high IgG levels, autoimmune hepatitis (AIH) was initially considered the leading diagnosis, supported by an International Autoimmune Hepatitis Group (IAIHG) score consistent with probable AIH. She was treated with intravenous methylprednisolone, followed by oral steroids; however, no clinical or biochemical improvement was observed. A liver biopsy was planned but was deferred because of her deteriorating condition in the pediatric intensive care unit.

Persistent bicytopenia and increasing transfusion requirements necessitated hematology consultation. A bedside bone marrow biopsy was attempted but yielded inconclusive results. Flow cytometric analysis of ascitic fluid revealed a clonal T‐cell population, consistent with T‐cell acute lymphoblastic leukemia (T‐ALL), confirming the final diagnosis. Immunophenotyping for flow cytometry yielded CD3 greater than CD8 positive T cells with sprinkling of CD7 positive in benign T lymphocytes.

Upon arrival at the emergency department, the initial emergency treatment focused on stabilization. She was started on oxygen at 5 L/min via a face mask, placed on fluid restriction, and commenced on maintenance intravenous fluids consisting of 0.9% dextrose‐saline at a rate of 38 mL/h (900 mL over 24 h). Oral intake was restricted, and intravenous ceftriaxone was initiated at a dose of 75 mg/kg/day in two divided doses (1275 mg twice daily) to cover potential bacterial infection. Her vital signs, abdominal girth, and intake/output were monitored frequently every 6 h.

Due to her markedly elevated ANA titer of 1:640, raised IgG levels of 22.53 g/L, and progressive hepatosplenomegaly, autoimmune hepatitis was suspected, and she was started on intravenous methylprednisolone at 2 mg/kg/day (40 mg/day) for treatment. This was transitioned to oral prednisolone at 1.5 mg/kg/day after a few days. Despite 20 days of corticosteroid therapy, there was no clinical or laboratory improvement, and plans for a diagnostic liver biopsy were deferred as her condition worsened in the pediatric intensive care unit (ICU).

Following the final diagnosis of T‐ALL Approximately 2 weeks after the failed corticosteroid therapy, the patient was promptly started on a pediatric T‐ALL induction chemotherapy protocol. The treatment included weekly vincristine at 1.5 mg/m^2^ and doxorubicin at 25 mg/m^2^ for 4 weeks, a single intravenous dose of pegaspargase at 2500 IU/m^2^, and daily oral prednisolone at 60 mg/m^2^ for 28 days, followed by a taper. Intrathecal methotrexate was administered weekly for central nervous system (CNS) prophylaxis. She tolerated induction therapy with supportive transfusions, infection prophylaxis, and nutritional support well. Minimal residual disease (MRD) testing of bone marrow aspirate was planned at the end of induction to guide subsequent risk‐adapted therapy.

## 4 Conclusion and Results (Outcomes and Follow‐Up)

After induction, the patient received high‐risk consolidation with cyclophosphamide, cytarabine, and oral 6‐mercaptopurine, followed by interim maintenance with methotrexate, peg‐asparaginase, and vincristine. Delayed intensification consists of reinduction and reconsolidation with doxorubicin, vincristine, PEG‐asparaginase, dexamethasone, cytarabine, and cyclophosphamide. Maintenance treatment will be continued for 2 years with daily 6‐mercaptopurine, weekly methotrexate, monthly vincristine, pulses of dexamethasone, and intrathecal methotrexate. The patient is currently undergoing induction therapy under close supervision.

This case highlights the challenges in diagnosing pediatric T‐cell Acute Lymphoblastic Leukemia (T‐ALL), particularly when it presents atypically with ascites and minimal classic symptoms such as bone marrow infiltration, CNS involvement, and an enlarged thymus. The diagnosis was delayed due to the initial suspicion of an autoimmune disease and an inconclusive bone marrow biopsy, with the help of ascitic fluid flow cytometry. Eventually, T‐ALL cells were observed, and the diagnosis was confirmed. This highlights the value of varied and thorough diagnostic approaches and advanced testing when standard methods fail. Clinicians should maintain a high index of suspicion for malignancy in children with persistent and unexplained symptoms. The patient is currently receiving protocol‐based chemotherapy for T‐ALL, emphasizing the importance of timely diagnosis and specialized care to improve outcomes.

## 5 Discussion

Acute Lymphoblastic Leukemia (ALL) is a malignancy that affects the blood and bone marrow. It is characterized by the rapid and uncontrolled growth of immature lymphocytes, known as lymphoblasts. These abnormal cells proliferate extensively. The inhibition of normal blood cell formation results in the typical signs of leukemia, such as anemia, thrombocytopenia, and the generation of ineffective white blood cells that increases vulnerability to infections [7]. In pediatric patients, T‐cell acute lymphoblastic leukemia (T‐ALL) accounts for about 12%–15% of diagnosed ALL cases. Historically, T‐ALL had a significantly poorer prognosis compared to B‐ALL, but recent advancements in treatment have led to improved survival rates. Moreover, the cure requires an extensive amount of therapies [8]. Treatment for childhood ALL typically involves a multi‐phase chemotherapy regimen, extending over a period of 2–3 years, a comprehensive protocol that includes distinct phases: induction, consolidation, interim maintenance, delayed intensification, and long‐term maintenance [7, 9].

The induction phase is designed to achieve rapid action by eliminating most leukemic blasts. The regimen for this patient included weekly vincristine and doxorubicin for 4 weeks, Peg L‐asparaginase, prednisolone/dexamethasone, and intrathecal methotrexate. This combination is consistent with the standard induction regimens for T‐ALL. By administering intrathecal methotrexate during this phase, the importance of early central nervous system (CNS) prophylaxis in all cases of ALL is emphasized, especially in TALL, which is more likely to involve the CNS. Consolidation is the immediate step after induction of chemotherapy; the purpose of this phase is to erase any remaining leukemia cells that might have survived the initial treatment. The chemotherapeutics planned for this phase include cyclophosphamide, cytarabine, and 6‐mercaptopurine (6 MP), which are commonly established consolidation regimens. The interim phase after maintenance involves less intense chemotherapy courses designed to prevent relapse between the more intensive phases. In our case report, the plan specifies methotrexate, Peg‐asparaginase, and vincristine (IM Capizzi), a standard component of many ALL protocols. Delayed Intensification is more like a re‐induction and re‐consolidation phase, utilizing a potent combination of doxorubicin, vincristine, Peg‐asparaginase, dexamethasone, cytarabine, and cyclophosphamide. This is a high‐intensity phase crucial for preventing the recurrence of the disease.

Finally, the last phase is the maintenance phase, which is a long‐term phase in which lower‐intensity therapy is designed to sustain and prevent late relapses. The patient's prescribed regimen included 2 years of 6 MP and methotrexate, along with intrathecal methotrexate, monthly vincristine, and pulsed dexamethasone. This aligns with the typical 2–3‐year duration for maintenance therapy in ALL [7, 9].

Flow cytometry is also a highly effective tool for the diagnosis and classification of leukemia. For T‐cell ALL, markers such as CD2, CD3, CD4, CD5, and CD7 are key for characterization. Crucially, flow cytometry can be performed on different body fluids, including ascitic fluid. This is useful in cases where bone marrow biopsies are inconclusive or technically challenging to obtain. In this specific case, ascitic fluid was used. Flow cytometry directly facilitated the definitive diagnosis of T‐ALL, demonstrating the diagnostic power of modern immunophenotyping techniques in challenging clinical scenarios. It also shows that clinicians should consider alternative sample sources for flow cytometry when standard samples are uninformative, especially in cases with suspected extramedullary disease(happens when myeloma cells causes tumors in the body soft tissues or organs that are outside of the bone marrow), thereby reducing diagnostic delays and facilitating proper treatment [10, 11]. The table outlines the key immunophenotypic markers typically assessed via flow cytometry for T‐ALL.

The most common symptoms of acute lymphocytic leukemia are nonspecific and may be difficult to distinguish from common self‐limited diseases of childhood. In a meta‐analysis, more than half of the children with childhood leukemia had at least one of the following five features on presentation: palpable liver and spleen, pallor, pyrexia, or bruising. ALL patients typically present with symptoms of night sweats, easy bruising, pale skin, unexplained lymphadenopathy, weakness, weight loss, hepatosplenomegaly, or shortness of breath; however, others present with superior vena cava syndrome (obstruction of the superior vena cava), bone pain, mental changes, and oliguria (low urine output), testicular enlargement, musculoskeletal pain, mediastinal mass, and incidentally found peripheral blood cell abnormalities [12].

The pediatric leukemias can also present with non‐specific symptoms such as fever, fatigue, pallor (pale appearance), and organomegaly (enlargement of organs) [2]. Hepatosplenomegaly (enlargement of the spleen) is common in both cases [1]; however, the clinical presentation can deviate from these typical patterns, presenting as atypical manifestations such as intussusception, hypercalcemia, diffuse osteoporosis, and vertebral fracture, with no classical symptoms or signs on peripheral smear [1]. However, in some cases, the complete blood count revealed pancytopenia with blasts in the peripheral smear, with a history of multiple pathological fractures, generalized osteopenia, and vertebral compression [2]. Acute Lymphoblastic Leukemia (ALL) can also cause persistent hyper‐eosinophilia [13], and the diagnosis of pediatric cancers is frequently complicated by delays.

In this case, the patients presented with an atypical clinical picture since they presented with ascites (fluid buildup in the peritoneal cavity that causes abdominal swelling) and generalized edema (fluid accumulation that affects the whole body rather than particular organs or body areas), and also showed symptoms such as fever and hepatosplenomegaly. This atypicality, particularly when combined with the absence of common ALL symptoms such as bone pain, directly contributed to the initial diagnostic ambiguity and the exploration of non‐oncological differential diagnoses. There is a need for a higher suspicion of malignancy, even when a patient's symptoms initially suggest a more common or harmless pediatric condition. The patient's background, including poor socioeconomic status, consumption of unboiled water, and a family history of untreated tuberculosis, can influence the initial diagnostic considerations for infections such as disseminated tuberculosis. This illustrates how broader social health can shape and potentially prolong diagnostic pathways, especially in cases where symptoms are non‐specific.

## Author Contributions

**Kainat Shaikh:** conceptualization, data curation, formal analysis, investigation, methodology, project administration, resources, software, validation, writing – original draft, writing – review and editing. **Zahra Anas:** conceptualization, data curation, formal analysis, investigation, methodology, resources, software, validation, visualization, writing – original draft, writing – review and editing. **Samia Rajput:** conceptualization, data curation, formal analysis, funding acquisition, investigation, methodology, project administration, resources, visualization, writing – original draft, writing – review and editing. **Alizeh Zaib:** data curation, formal analysis, investigation, methodology, software, validation, writing – original draft, writing – review and editing. **Ayesha Saleem:** conceptualization, data curation, investigation, methodology, project administration, resources, software, validation, visualization, writing – original draft, writing – review and editing. **Md Ariful Haque:** formal analysis, funding acquisition, methodology, project administration, supervision, visualization, writing – review and editing.

## Funding

The authors have nothing to report.

## Ethics Statement

The authors have nothing to report.

## Consent

Written informed consent was obtained from the patient's parents/legal guardian for publication and any accompanying images. A copy of the written consent is available for review by the Editor‐in‐Chief of this journal on request.

## Conflicts of Interest

The authors declare no conflicts of interest.

## Data Availability

Data sharing is not applicable to this article as no new data were created or analyzed in this study.
